# Pediatric donor cell leukemia after allogeneic hematopoietic stem cell transplantation in AML patient from related donor

**DOI:** 10.1186/s13039-014-0105-4

**Published:** 2015-01-31

**Authors:** Lucina Bobadilla-Morales, Helia J Pimentel-Gutiérrez, Sergio Gallegos-Castorena, Jenny A Paniagua-Padilla, Citlalli Ortega-de-la-Torre, Fernando Sánchez-Zubieta, Rocio Silva-Cruz, Jorge R Corona-Rivera, Abraham Zepeda-Moreno, Oscar González-Ramella, Alfredo Corona-Rivera

**Affiliations:** Laboratorio de Citogenética, Genotoxicidad y Biomonitoreo, Instituto de Genética Humana “Dr. Enrique Corona Rivera”/Doctorado de Biología Molecular, Departamento de Biología Molecular y Genómica, Universidad de Guadalajara, Guadalajara, Jalisco México; Instituto de Investigación en Cáncer de la Infancia y la Adolescencia, Centro Universitario de Ciencias de la Salud, Universidad de Guadalajara, Guadalajara, Jalisco México; Unidad de Citogenética, Servicio de Hematología y Oncología Pediátrica, División de Pediatría, Nuevo Hospital Civil de Guadalajara, “Dr. Juan I. Menchaca”, Guadalajara, Jalisco México; Servicio de Hematología y Oncología Pediátrica, División de Pediatría, Nuevo Hospital Civil de Guadalajara, “Dr. Juan I. Menchaca”, Guadalajara, Jalisco México; División de Pediatría, Centro de Registro e Investigación sobre Anomalías Congénitas (CRIAC), Nuevo Hospital Civil de Guadalajara, “Dr. Juan I. Menchaca”, Guadalajara, Jalisco México; Laboratorio de Citogenética Genotoxicidad y Biomonitoreo, Instituto de Genética Humana “Dr. Enrique Corona-Rivera”, Departamento de Biología Molecular y Genómica, Centro Universitario de Ciencias de la Salud, Universidad de Guadalajara, #Sierra Mojada 950, S.L., Edificio P, Nivel 2, Col. Independencia, Guadalajara, Jalisco CP: 44340 México

**Keywords:** Allogeneic hematopoietic stem cell transplantation, Acute myeloid leukemia, Donor cell leukemia

## Abstract

Here we present a male patient with acute myeloid leukemia (AML) initially diagnosed as M5 and with karyotype 46,XY. After induction therapy, he underwent a HLA-matched allogeneic hematopoietic stem cell transplantation, and six years later he relapsed as AML M1 with an abnormal karyotype //47,XX,+10[2]/47,XX,+11[3]/48,XX,+10,+11[2]/46,XX[13]. Based on this, we tested the possibility of donor cell origin by FISH and molecular STR analysis. We found no evidence of Y chromosome presence by FISH and STR analysis consistent with the success of the allogeneic hematopoietic stem cell transplantation from the female donor. FISH studies confirmed trisomies and no evidence of MLL translocation either p53 or ATM deletion. Additionally 28 fusion common leukemia transcripts were evaluated by multiplex reverse transcriptase-polymerase chain reaction assay and were not rearranged. STR analysis showed a complete donor chimerism. Thus, donor cell leukemia (DCL) was concluded, being essential the use of cytological and molecular approaches. Pediatric DCL is uncommon, our patient seems to be the sixth case and additionally it presented a late donor cell leukemia appearance. Different extrinsic and intrinsic mechanisms have been considered to explain this uncommon finding as well as the implications to the patient.

## Background

Acute myeloid leukemia (AML) is a clinically and genetically heterogeneous hematological disease. Patient age, cytogenetic and minimal residual disease after induction therapy, play a major role in the classification and prognosis of AML, leading to stratification in risk categories [1,2].

Allogeneic hematopoietic stem cell transplantation (alloHSCT), results in superior disease-free survival and overall survival (OS) rates in patients with intermediate and high risk AML. Nowadays, many progresses have been made concerning donor selection, reduction of myeloablative conditioning and management of the immunologic graft-versus-leukemia effect that all together provides a therapeutic effectiveness. Nevertheless all these advances, leukemia relapse remains the main problem that reduces the cure rates after alloHSCT [3]. A relapse is diagnosed when patient-derived cells survived the myeloablative therapy and the original leukemic cell reappears at any site in the body. Most of them occur during the first year of treatment, late relapse is not common and occurs after two years of treatment. Secondary leukemia may develop after successful treatment when non-biologically related clone to original cells arise and appears as hematologic neoplasia. Secondary leukemia must be differentiated from late relapse which descend from the same original cell that originated the primary malignancy [4]. Secondary leukemia can develop in a patient-derived clone, but in some rare cases, arise from engrafted donor cells [5], and becomes a new disorder known as donor cell leukemia (DCL). DCL was described in 1971 by Fialkow and colleagues and through 2011, more than 80 cases have been reported in the literature [6-8]. Most of the reported secondary AML arise after an autologous transplantation, and not allogeneic transplantation [4], this might be due to the lack of graft versus leukemia effect [5]. Relapse is observed in at least 33% of the transplanted patients with AML/myelodysplastic syndrome and Wiseman *et al.* [6], suggested that up to 5% of all post-transplant leukemia correspond to DCL. Different mechanisms have been proposed to explain the etiology of DCL [9], however it remains to be established if the original mechanism whether occurred in donor or receptor cells. Most of the reported cases arise from adult patients. Pediatric DCL is uncommon, our patient seems to be the sixth reported case presented with a late appearance after 68 months. The aim of the present report is to present a male boy with AML-M5 at diagnosis successfully treated with HLA-matched sibling alloHSCT who developed a second AML-M1 in cells of donor origin.

## Case presentation

**Figure 1:**
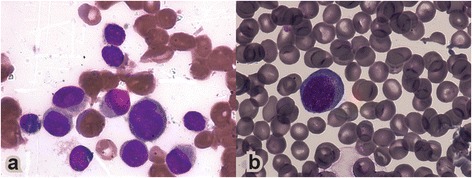
**Bone marrow aspirates. a)** At diagnosis as LMA M5. **b)** At relapse as LMA M1.

A 9-year-old boy was diagnosed with AML-M5 by French American British (FAB) classification in December 2005. The bone marrow aspirate showed 100% blasts and cerebrospinal fluid was positive for leukemic blasts. Cytogenetic analysis showed a normal karyotype 46,XY. Flow cytometry immunophenotype was positive to CD13, CD33, CD34, CD38, HLA-DR, MPO, CD14, and CD11. The treatment consisted of two cycles of cytarabine 1000 mg/m2 three weeks apart. Minimal residual disease was positive at day 14 and two more induction cycles were given to achieve complete remission. He received consolidation chemotherapy and was successfully treated with alloHSCT transplantation from his HLA-identical sister. Conditioning regimen consisted on busulfan 16 doses of 1 mg/kg/PO every 6 hours and cyclophosphamide 120 mg/kg. Sixty-eight months after transplant the patient presented febrile with neutropenia and thrombocytopenia, bone marrow aspirates showed 98% of blasts consistent with AML M1 (Figure 1a-b), which was compatible with the immunophenotype.

The patient died after two induction cycles with the National Myeloid Protocol which consist of 7 doses of cytarabine 100 mg/m2, 3 doses of daunorrubicin 50 mg/m2 and five days with central nervous system prophylaxis with intrathecal chemotherapy (methotrexate/cytarabine/hydrocortisone). Minimal residual disease before the second cycle was 8% and the patient died by septic shock 7 days after he finished the second cycle.

The bone marrow morphology of M5 at diagnosis was confirmed with immunophenotype, CD13, CD33, CD34, CD38, HLA-DR, MPO, CD14, and CD11. At relapse, the morphology and immunophenotype positive for HLA-DR, CD117, MPO, CD38, CD34, CD45, CD15 (mild) and CD13, concluded AML M1. Additional studies were performed on relapse as follows.

## Discussion and conclusions

Here we present a case of a DCL AML-M1 formerly diagnosed as AML-M5 in a pediatric male patient successfully treated with a HLA-matched alloHSCT. The karyotype, FISH and STR analysis evidenced a completely new disease on female donor cells, indicating a successful transplant and implying a different and more complex approach of the case.

DCL is a rare but well-recognized disease that affects patients treated with hematopoietic cells transplantation as a late complication [6]. This patients develop a secondary leukemia from cells derived from the donor and the diagnosis depends on the ability to accurately identify the donor origin of the leukemic cells [8]. In these case we conclude that our patient acquired a secondary leukemia derived from his sister donor cells since FISH analysis showed 100% of XX centromeres of the analyzed leukocytes. Molecular testing by STR analysis demonstrated that sample from bone marrow and mucosal swap from the patient had different DNA.

Abnormal cytogenetics has been found in 72% of cases with DCL/myelodisplastic neoplasms, being the most common aberrations chromosome 7 monosomy or 7q deletion (29%), occurring always in myeloid neoplasms (AML or myelodysplastic syndrome (MDS) so far there is no evidence of a structural or numerical aberration patterns in DCL [7,10]. The karyotype may be the initial approach to search for DCL [6], as occurred in our patient. Isolated trisomy 10 is an extremely uncommon event, in pediatric leukemia. In a review, Yuan et al., reported just 3 pediatric and 20 adults AML patients with trisomy 10 and their impact in disease progression and prognosis remains unclear [11]. Despite the few cases with +10, the most have been observed in AML M1 patients [12]. Trisomy 11 is a rare event associated with poor prognosis, Alseraye et al. [1], reported a group of 18 patients with trisomy 11 that included *de novo* AML cases, patients with a history of MDS and patients with a progressive clinical course of refractory or relapsed disease. Suzuki et al. [12], found a low incidence of trisomy 10 (0.28%) and trisomy 11 (0.47%) as a sole abnormality from 1074 adult AML patients. Morphology of 55% of the patients was classified as AML-M1 and the OS was 5 months. We did not find a case report with both 10 and 11 trisomies in the same patient, in concordance with Alseraye et al. [1], our patient classified as AML-M1 and trisomy 11, also presented an unfortunate ending since he died before acquire remission after 2 months of diagnosis from the DCL. The initial and post-transplant karyotypes suggest that the original leukemic cell population was different from the new one. Furthermore, TEL-AML1 fusion gene, which has been implicated in late relapses [13], was not translocated in our patient [13]. Our case did not have structural abnormalities and observed numerical aberrations seem to be associated to the observed leukemia, at present no prognosis abnormality has been defined.

Previous reports have shown that alkylating agents, and topoisomerasa II inhibitors are a risk factor to develop secondary AML [14]. Patients treated with topoisomerasa-II inhibitors typically results with MLL (11q23) [15], or less common RUNX1 gene (21q22) translocations. Alkylating agents presents in 90% of the cases loss of part or complete chromosomes 5 and/or 7 [14,16]. Our patient was treated with doxorubicin a topoisomerasa-II inhibitor and cyclophosphamide, a well known alkylating agent [14] and due to the treatment received, the patient was in the higher risk to develop a secondary leukemia however, we did not find any rearrangement in MLL gene or deletion in chromosome 5 and 7. Then, we may no support in our patient chemotherapy related DCL.

Wiseman [6], reported that 75% of DCL are different from the original disease and that arise as AML in 53% of cases 25% as ALL and 20% as MDS. Our patient was formerly diagnosed with AML-M5 and as with AML-M1 in the second leukemia, maintaining the same lineage but in a different maturation stage. We found in the literature, 5 pediatrics cases of DCL. In 3/5, they relapsed with different lineage and in contrast with the adult DCL which most of them originates in an AML or MDS, in the pediatric patients 50% of the DCL are ALL and 50% AML. In previous reports of pediatrics patients, the maximum period of time after transplant and DCL was 23 months, in our case the patient relapsed after 68 months. The pediatric DCL cases are presented in Table 1.

**Table 1:** **Reported pediatric cases of donor cell leukemia and present case**

**Age**	**1st disease**	**2nd disease**	**Months after transplant**	**Source**	**Donor**	**Author**
4	AML	AML	23	BM	Brother	[17]
12	CML	ALL	15	BM	Sister	[18]
NA*	T-ALL	B-ALL	NA	BMT	NA	[19]
16	AML	B-ALL	12	BMT	Brother	[20]
3	AML M5	AML	14.5	UCB	Unrelated	[7]
16	AML M5	AML M1	68	BMT	Sister	Present case

NA* = Not Available.

Different mechanisms have been proposed to explain the etiology of DCL. Extrinsic factors focus on chemotherapy and radiation-induced stromal abnormalities [6,8,21]. Intrinsic factors may be related to the donor, such as leukemia previously present in donor cells or leukemic predisposition [6]. On the other hand intrinsic factors can be related to the recipient, including impaired immune function [8], transformation of donor cells by antigenic stimulation through host tissue, oncogene transfection from abnormal to normal cells [9], replication stress, and host microenvironment [7,22]. Not all cases that develop DCL arise from previous malignant diseases [9], suggesting alternative explanations to stromal abnormalities and oncogene transfection theories [9,10]. Any specific conditioning, graft manipulation and graft-versus-host disease prophylaxis has been identified as risk factors for DCL [23].

Additionally to numerical chromosomal abnormalities, we were not able to detect structural cytogenetic and molecular abnormalities in donor cells. Related to this, the donor sister has been maintained without clinical evidence of hematologic disorder, and apparently healthy. Besides, the observation of normal tested tumor suppressor genes, fusion genes absence, and MLL integrity, support that more host microenvironment conditions that donor cells may be implicated. Despite the microenvironmental theory, more information about the disease biology is needed to confirm if the original mechanism occurred in donor or receptor cells [9,21,24]. A multifactorial component may be involved, and considering that from 5/6 pediatric DCL the donor has been related, there could be a familial predisposing involvement.

In summary, pediatric DCL might be a less heterogeneous group that those observed in adults. To identify the presence of DCL is essential the use of cytological and molecular approaches which eliminate the possibility of a relapse from the original clone. Nevertheless the DCL in our patient has been well supported, the direct mechanism involved is still elusive. We suggest that defects in the host marrow microenvironment may be frequently implicated.

## Material and methods

### Cytogenetic studies

**Figure 2:**
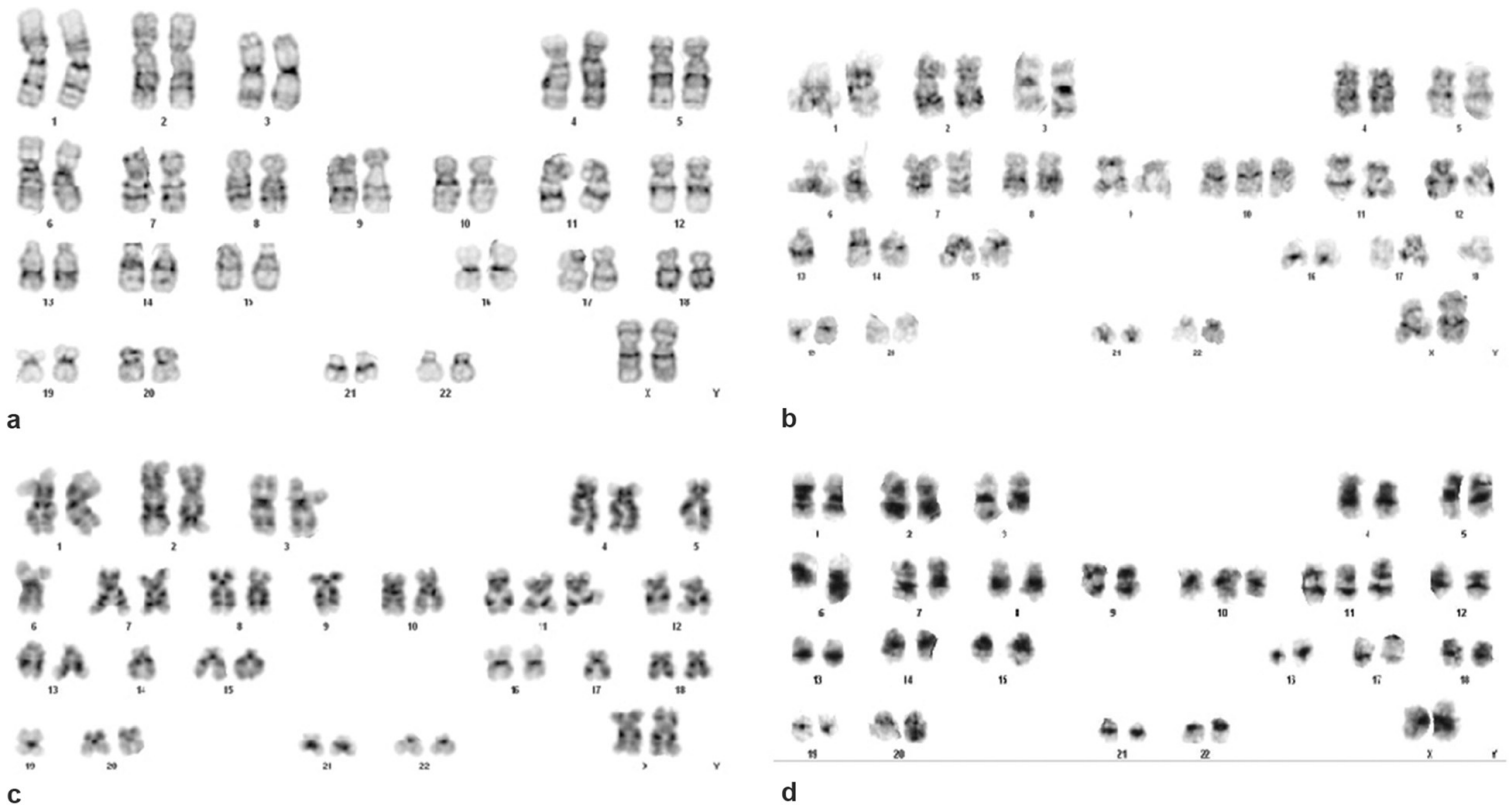
**Karyograms from the patient at relapse. a)** Normal metaphase. **b)** Trisomy of chromosome 10. **c)** Trisomy of chromosome 11. **d)** Trisomies of chromosomes 10 and 11.

Cytogenetic analysis performed on bone marrow sample using standard culture methods and GTW banding showed an abnormal female karyotype: //47,XX,+10[2]/47,XX,+11[3]/48,XX,+10,+11[2]/46,XX[13] (Figure 2a-d).

### FISH studies

To test for the presence of additional 10 and 11 chromosomes, we performed Fluorescence *in situ* hybridization (FISH), on bone marrow samples using centromere 10 (CEP 10 green, KBI-20010G, Kreatech), and MLL (LSI dual dolor break apart, 05 J90-001, Abbott Molecular) FISH probes. The results were reported according to ISCN 2013 as follows. Centromere 10: //nucish(D10Z1x3)[92/200]. MLL: //nuc ish(5′MLL con 3′MLLx3)[150/200]. Cep 10 and MLL showed three copies, agreeing with the trisomy 10 and 11 found in karyotype (Figure 2b,c).

**Figure 3:**
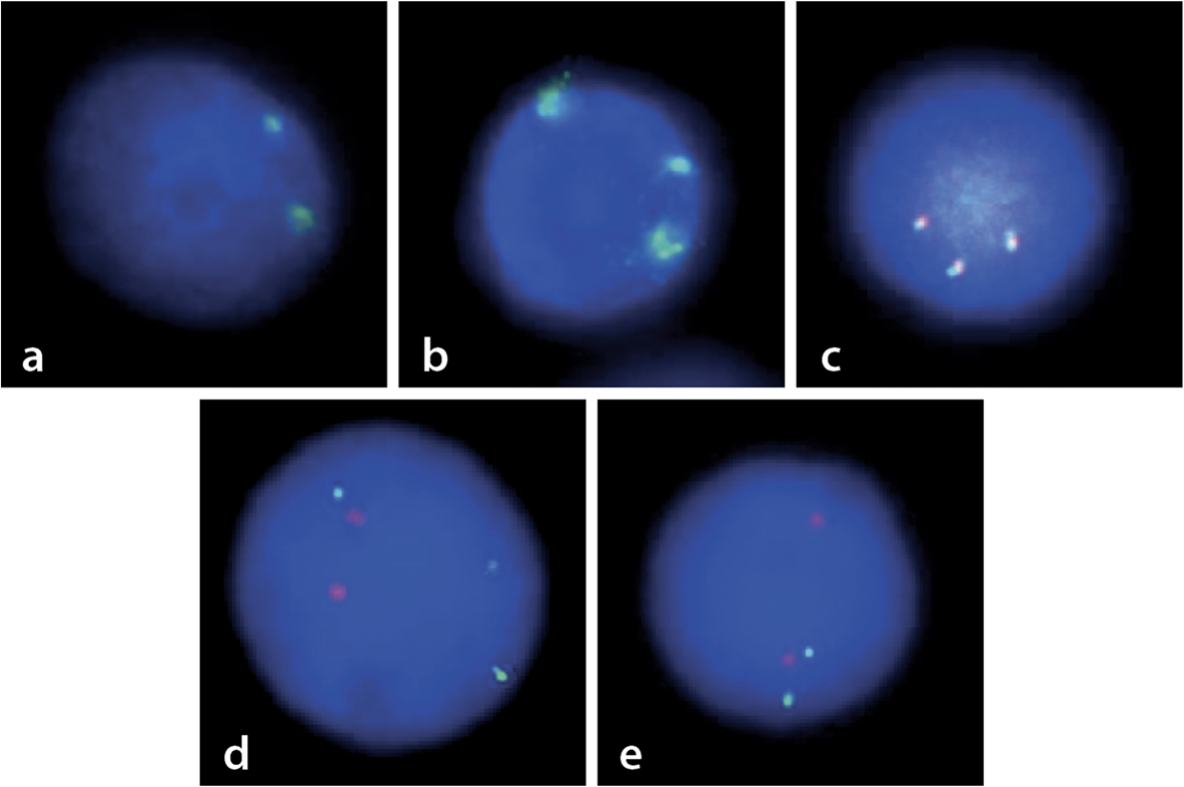
**Interphase FISH studies. a)** XY probe (X spectrum green and Y spectrum red) shows the presence of donor female cells. **b)** CEP 10 (spectrum green) probe supports the trisomy of chromosome 10. **c)** No disruption of MLL gene was observed using a MLL break apart probe; the third signal corresponds to the extra chromosome 11. **d)** Simultaneous p53 (spectrum green) and ATM (spectrum Orange) test, showed no deletion of any suppressor gene, but the third signal of ATM agrees with the trisomy 11. **e)** Tel-AML1 (red/green signal) evaluation was negative for the presence of the fusion gene.

The sex chromosomes status was tested using centromeres X/Y (SE DXZ1 green/SE DXZ1 red, KBI-20030, Kreatech) FISH probe, resulting as follows: //nucish(DXZ1x2)[192/200]. The presence of a pair of centromere X sequences supports a donor cell origin, (Figure 3a).

Complimentary FISH probes on bone marrow samples using TEL/AML1(LSI TEL SG/LSI AML1 SO, 05 J62-001, Abbott Molecular) and TP53/ATM (LSI TP53 SG/LSI ATM SO, 05 J83-001, Abbott Molecular), resulted in normal fluorescence patterns, although ATM exhibited an additional signal showing the extra 11 chromosome (Figure 3d,e).

*STR studies.* Microsatellite short tandem repeat (STR) analysis were performed for 15 loci (CSF1PO, D2S1338, D3S1358, D5S818, D7S820, D8S1179, D13S317, D16S539, D18S51, D19S433, D21S11, FGA, TH01, TPOX, and vWA) and sex determination by amelogenin (AmpFLSTR Identifiler, 4322288, Life Technologies) on genomic DNA from bone marrow and mucosal swap of the post-transplant patient and in blood from the donor (Table 2). From the 15 microsatellite markers tested, differences were found between the oral mucosal and blood from the patient in the 9/14 informative alleles and Y chromosome was not detected concluding that the AML was derived from the donor cells.

**Table 2:** **Post-transplant STR analysis from the patient mucosal swap, bone marrow, and blood from the donor**

**Marker**	**Patient’s mucosal swap at relapse**		**Patient’s bone marrow at relapse**		**Donor’s blood**	
D8S1179	10	10	10	10	10	10
D21S11	28	30	28	30	28	30
D7S820	10	12	10	12	10	12
CSF1PO*	**9**	**12**	**9**	**10**	**9**	**10**
D3S1358*	**15**	**17**	**17**	**17**	**17**	**17**
TH01*	**7**	**7**	**8**	**8**	**8**	**8**
D13S317*	**10**	**13**	**10**	**12**	**10**	**12**
D16S539*	**10**	**12**	**10**	**10**	**10**	**10**
D19S433	14.2	15	14.2	15	14.2	15
vWA*	**16**	**16**	**16**	**18**	**16**	**18**
TPOX*	**8**	**12**	**11**	**11**	**11**	**11**
D18S51	17	18	17	18	17	18
DS5818*	**7**	**12**	**12**	**12**	**12**	**12**
FGA*	**24**	**25**	**25**	**26**	**25**	**26**
Amelogenina	X	Y	X	X	X	X

* = informative alleles.

### Multiplex PCR studies

To detect the most common fusion transcripts found in AML and ALL, we performed nested multiplex reverse transcriptase-polymerase chain reaction assay (HemaVision, HV01-28 N, DNA Technology A/S). The 28 fusion transcripts evaluated are shown in Table 3. Our patient was negative for the amplification of all of them, while the endogenous controls were positive in every PCR reaction.

**Table 3:** **Involved translocation genes studied by nested multiplex PCR**

Traslocation*	**Genes**	**Traslocation**	**Genes**	**Traslocation**	**Genes**
t(X;11)(q13;q23)	MLL (11q23)	TAL1 (deleción)	STIL (1p32)	t(9;22)(q34;q11)	BCR (22q11)
	FOZO4 (Xq13.1)		TAL1 (1p32)		ABL1 (9q34.1)
t(6;11)(q27;q23)	MLL (11q23)	t(8;21)(q22;q22)	RUNX1 (21q22.3)	t(9;12)(q34;p13)	ETV6 (12p13)
	MLLT4 (6q27)		RUNX1T1 (8q22)		ABL1 (9q34.1)
t(11;19)(q23;p13.1)	MLL (11q23)	t(3;21)(q26;q22)	RUNX1 (21q22.3)	t(5;12)(q33;p13)	ETV6 (12p13)
	ELL (19p13.1)		MDS1 (3q26)		PDGFRB (5q33)
t(10;11)(p12;q23)	MLL (11q23)	t(16;21)(p11;q22)	FUS (16p11.2)	t(12;22)(p13;q11-12)	ETV6 (12p13)
	MLLT10(10P12)		ERG (21q22.3)		MN1 (22q12.1)
t(1;11)(p32;q23)	MLL (11q23)	t(15;17)(q22;q12)	PML (15q22)	t(6;9)(p23;q34)	DEK (6p23)
	EPS15 (1p32)		RARA (17q12)		NUP214 (9q34)
t(11;17)(q23;q12-21)	MLL (11q23)	t(9;22)(q34;q11)	BCR (22q11)	t(9;9)(q34;q34)	SET (9q34)
	MLLT6 (17q21)		ABL1 (9q34.1)		NUP214 (9q34)
t(11;19)(q23;p13.3)	MLL (11q23)	t(4;11)(q21;q23)	MLL (11q23)	inv(16)(p13;q22)	CBFB (16q22.1)
	MLLT1 (19P13.3)		AFF1 (4q21.3)		MYH11 (16p13.11
t(10;11)(p12;q23)	MLL (11q23)	t(10;11)(p12;q23)	MLL (11q23)	t(3;21)(q26;q22)	RUNX1 (21q22.3)
	MLLT10 (10p12)		MLLT10 (10p12)		EAP (3q26)
t(9;11)(p22;q23)	MLL (11q23)	t(11;19)(q23;P13.3)	MLL (11q23)	t(11;17)(q23;q12-21)	ZBTB16 (11q23)
	MLLT3 (9p22)		MLLT1 (19p13.3)		RARA (17q12)
t(1;19)(q23;p13)	TCF3 (19p13.3)	t(9;11)(p22;q23)	MLL (11q23)	t(3;21)(q26;q22)	RUNX1 (21q22.3)
	PBX1 (1q23.3)		MLLT3 (9p22)		EVI1 (3q26)
t(17;19)(q22;p13)	TCF3 (19p13.3)	t(1;11)(q21;q23)	MLL (11q23)	t(15;17)(q22;q12)	PML (15q22)
	HLF (17q22)		MLLT11 (1q21)		RARA (17q12)
t(12;21)(p13;q22)	ETV6 (12p13)	inv(16)(p13;q22)	CBFB (16q22.1)	t(3;5)(q25.1;q35)	NPM1 (5q22)
	RUNX1 (21q22.3)		MYH11 (16p13.11		MLF1 (3q25.1)

* = All the translocations tested were negative in our patient.

## Consent

Written informed consent was obtained from the patient's parents for publication of this Case report and any accompanying images. A copy of the written consent is available for review by the Editor-in-Chief of this journal.

## Abbreviations

AML: Acute myeloblastic leukemia
alloHSCT: Hematopoietic stem cell transplant
FAB: French American British
FISH: Fluorescence *in situ* hybridization
GTW: G bands by trypsin and Wright’s stain
HLA: Human leukocyte antigen
MDS: Myelodysplastic syndrome
nucish: Nuclear *in situ* hybridization
OS: Overall survival
STR: Short tandem repeats
UCB: Umbilical cord blood
